# Supplementary material for: Accuracy of a deep convolutional neural network in the detection of myopic macular diseases using swept-source optical coherence tomography
Source: PLoS One. 2020 Apr 16;15(4):e0227240. doi: 10.1371/journal.pone.0227240 (PMC7161961; doi:10.1371/journal.pone.0227240)
Supplement: Attachment — Submitted filename: Response to Revierers.docx [file pone.0227240.s002.docx]

We appreciate the careful reviews and instructive suggestions from the reviewers. We have revised our manuscript following your suggestions. In the course of doing so, we discovered incorrect word in Table 4. And we have added patients with comorbidities (mild cat, epiretinal membrane, chorioretinal atrophy, macular hole and so on) to the nHM and HM groups that mimic those encountered in real life, as you and the reviewer kindly pointed out. And the sentence “Some nHM and HM images included with comorbidities (mild cat, epiretinal membrane, macular hole and so on).” has been added (page7, lines100-102). Also, the sentences “Third, the DL performed in this study, though it showed high classification performance results, was limited to yielding only three types of classification. However, HM is often associated with various complications such as chorioretinal atrophy and macular hole retinal detachment. Therefore, it is necessary to train a network using a large dataset of HM fundus images with other complications.”have been deleted (Page 24, Line 368). Furthermore, as you advised, we added comorbidities images and re-analyzed. Therefore, the result data has changed slightly (All are highlighted).

We hope you will accept these changes. Our responses to the editor and reviewers are as follows:

*Journal Requirements:*

*1. When submitting your revision, we need you to address these additional requirements.*

*Please ensure that your manuscript meets PLOS ONE's style requirements, including those for file naming. The PLOS ONE style templates can be found at*

*http://www.journals.plos.org/plosone/s/file?id=wjVg/PLOSOne_formatting_sample_main_body.pdf and http://www.journals.plos.org/plosone/s/file?id=ba62/PLOSOne_formatting_sample_title_authors_affiliations.pdf*

**Reply:** Thank you for your suggestion. We confirmed that our revised manuscript meets PLOS ONE’s style.

*2. Please include in your financial disclosure statement the name of the funders of this study (as well as grant numbers if available). If your study was unfunded, please revise your financial disclosure statement to “The author(s) received no specific funding for this work.*

**Reply:** Thank you for your suggestion. The authors received no specific funding for this work. As your pointed out, the sentence “The authors received no specific funding for this work” has been added in the financial disclosure section of the cover letter.

*3. Thank you for stating the following financial disclosure:*

*The funders had no role in study design, data collection and analysis, decision to publish, or preparation of the manuscript.*

*a) Please provide an amended Funding Statement that declares *all* the funding or* *sources of support received during this specific study (whether external or internal to your organization) as detailed online in our guide for authors at http://journals.plos.org/plosone/s/submit-now.*

*b) Please state what role the funders took in the study. If any authors received a salary from any of your funders, please state which authors and which funder. If the funders had no role, please state: "The funders had no role in study design, data collection and analysis, decision to publish, or preparation of the manuscript."*

*Please include your amended statements within your cover letter; we will change the online submission form on your behalf.*

**Reply:** Thank you for your helpful suggestion. The authors received no specific funding for this work. As your pointed out, the sentence “The funders had no role in study design, data collection and analysis, decision to publish, or preparation of the manuscript.” has been added in the financial disclosure section of the cover letter.

*Reviewers' comments:*

*Review Comments to the Author*

*Reviewer #1: 1. Finacial Disclosure is missing:Although it is implied that this study was funded as per the phrase 'the funders had no role in stud design, decision to publish or interpretation of the manuscript', the funding source is not mentioned.*

**Reply:** Thank you for your helpful suggestion. As your and our editor pointed out, the sentences “The authors received no specific funding for this work. The funders had no role in study design, data collection and analysis, decision to publish, or preparation of the manuscript.” have been added in the financial disclosure section of the cover letter.

*2. The use of heatmaps to show areas of SS-OCT that the neural network focused compared to where retina specialists focused is very interesting.*

**Reply**: Thank you for your comment. We think heat maps is very helpful to know how AI diagnosis.

*3. Although the results of this study seem promising for Depp Learning, significant limitations still apply such as:*

*A. As mentioned in the manuscript, 'the original SS-OCT image size was 1,038 x 802 pixels but it was resized to 256 x 192 pixels to shorten the analysis time'*

**Reply**: We agree your suggestion. Your suggestion is the objective limitation of our study. As your pointed out, the sentence “Third, to shorten the analysis time, the original SS-OCT image with 1,038 × 802 pixels was resized to 256 × 192 pixels.” has been added (page24, lines 368-369).

*B. The exclusion of eyes with any other ocular coomrbidities including very common ones such as lens opacification, that favours DL as opposed to Retina Specialsts. The major limitation of this study is the design of DL in evaluating a single condition at a time, excluding eyes with coexisting retinal diseases. Improvement in this aspect is needed before DL could potentially be used in real life clinical settings.*

**Reply**: Thank you for your helpful suggestions. As your pointed out, we have added patients with comorbidities (mild cat, epiretinal membrane, chorioretinal atrophy, macular hole and so on) to the nHM and HM groups that mimic those encountered in real life. And we analyzed again including additional images. Thus, we rewrote Table 1. And the sentence “Some nHM and HM images included with comorbidities (mild cat, epiretinal membrane, chorioretinal atrophy, macular hole and so on). ”has been added (page7, line100-102).

*C. It would be interesting to train a DL network pass the limited binary or ternary classification and compare results accuracy with that of human Ophthalmologists.*

**Reply**: We are glad you are interested in our analysis method in this study.

*4. In the discussion, it is mentioned the ' in the present study, the DL model we used was able to obtain the same correct answer rate in a shorter time relative to Ophthalmologists using the same sample data', yet both in methods and results of the present study such comparisons of time are missing.*

**Reply**: Thank you for your suggestion. This is our mistake. According to this comment, the sentence “in a shorter time” has been deleted (Page 23, Line 348).

*5. In the baseline characteristics, AL and sex may be unavoidable to a certain extent in this type of study, yet the groups could have been matched in terms of age. There are significant differences in the age among the 4 groups of the present study p<0.001*

**Reply**: Thank you for your helpful comment. We agree that age adjustment is necessary. However, the patients with retinoschisis and mCNV increase with age, and the age could not be adjusted to secure a sufficient number of cases. If we can increase the number of target patients like a conducting joint research with other institutions, we think that we can adjust the age and lead to high-quality results. Please let us consider it for future study.

6. line 133: please correct 'itthe'

**Reply**: Thank you for your comment. the word “itthe” have been changed to “it the” (Page 9, Lines 134).
