# Supplementary material for: Accuracy of a deep convolutional neural network in the detection of myopic macular diseases using swept-source optical coherence tomography
Source: PLoS One. 2020 Apr 16;15(4):e0227240. doi: 10.1371/journal.pone.0227240 (PMC7161961; doi:10.1371/journal.pone.0227240)
Supplement: Attachment — Submitted filename: Response to Revierer.docx [file pone.0227240.s003.docx]

Editor,

PLoS ONE

March 22nd, 2020

Re: PONE-D-19-34572R1 entitled " Accuracy of a deep convolutional neural network in the detection of myopic macular diseases using swept-source optical coherence tomography".

To the Editor,

Thank you for your letter of March 20th, 2020 and for sending us the referee’s comments on our manuscript. We are returning the manuscript revised according to the comments.

We have studied the comments carefully and have made corrections which we hope meet with your approval. In the revised manuscript, all changes have been highlighted in yellow. Each of the coauthors has seen and agreed with each of the changes made to this manuscript in the revision.

Reviewer:

6. Review Comments to the Author

*Please use the space provided to explain your answers to the questions above. You may also include additional comments for the author, including concerns about dual publication, research ethics, or publication ethics. (Please upload your review as an attachment if it exceeds 20,000 characters)*

*Reviewer #1: One additional comment:*

*In Machine Learning and Deep Learning, a major concern and current limitation of AI algorithms created via training sets obtained using only one imaging device is the extent of generalizability to other commercially available similar imaging devices. In this study, the Topcon DRI OCT-1 was used for all OCT images. The authors should state in their limitations section that the AI algorithms created and tested herein might not be generelizable to other commercially available similar imaging devices - the extent of generalizability across different devices is yet to be investigated.*

Reply: Thank you for your helpful comment. As your pointed out, the sentences “Third, the AI algorithms created and tested herein might not be generalizable to other commercially available similar imaging devices, because we investigated by only the Topcon DRI OCT-1.” have been added in the limitations section of the manuscript (Page 24, Lines 368-370).

*Lines 311-314: the respective reference needs to be cited in the manuscript text.*

**Reply**: Thank you for your comment. It is our mistake. We added the respective reference number [49] in the manuscript text (Page 21, Line 311).
